# Comparing clinical and imaging features of patients with MOG antibody-positivity and with and without oligoclonal bands

**DOI:** 10.3389/fimmu.2023.1211776

**Published:** 2023-07-13

**Authors:** Yuji Tomizawa, Yasunobu Hoshino, Ryota Kamo, Davide Cossu, Kazumasa Yokoyama, Nobutaka Hattori

**Affiliations:** ^1^ Department of Neurology, School of Medicine, Juntendo University, Tokyo, Japan; ^2^ Department of Biomedical Sciences, Sassari University, Sassari, Italy; ^3^ Tousei Center for Neurological Diseases, Shizuoka, Japan

**Keywords:** myelin oligodendrocyte glycoprotein antibody-associated disease, oligoclonal bands, multiple sclerosis, relapse rate, IgG index

## Abstract

**Introduction:**

Myelin-oligodendrocyte glycoprotein antibody (MOG)–associated disorder (MOGAD) is a recently identified immune-mediated inflammatory disorder of the central nervous system (CNS). The significance of oligoclonal bands (OCBs) is not fully elucidated. This study investigated the clinical differences between patients with MOGAD who tested positive or negative for OCBs.

**Methods:**

The study was conducted on 23 patients with MOG-IgG-seropositivity who presented with central nervous system (CNS) symptoms. The patients were screened and divided into OCB-positive (n=10) and OCB-negative (n=13) groups, and their demographic, clinical, and magnetic resonance imaging (MRI) features were compared.

**Results:**

The results revealed that patients with OCB-positivity had a significantly higher frequency of relapse, and their IgG index was significantly higher.

**Discussion:**

OCBs were common in MOGAD met the consensus criteria. The study concluded that careful treatment decision-making is necessary in MOG antibody-positive cases with OCB-positivity.

## 1 Introduction

Myelin-oligodendrocyte glycoprotein (MOG) antibody-associated disorder (MOGAD) is a recently identified immune-mediated inflammatory disorder of the central nervous system (CNS). Diagnostic criteria for MOGAD have recently been proposed, further clarifying the disease entity (1). Among these criteria is the presence of oligoclonal bands (OCBs), which also strongly indicates the diagnosis of multiple sclerosis (MS). However, the occurrence of OCB-positive cases among patients who test positive for MOG antibodies is common (2, 3). OCBs are clonal immunoglobulins unique to the cerebrospinal fluid (CSF), and their presence indicates an immune response within the central nervous system. In MS, which is another immune-mediated CNS disease, oligoclonal bands are established as useful for diagnosis (4), whereas in MOGAD, the positivity rate of oligoclonal bands is reported to be about 10% (2). Therefore, the positivity of oligoclonal bands can be a factor that makes the differentiation between MOGAD and MS unclear. In MS positive OCBs are associated with worse survival outcomes (5–7). The significance of OCBs in patients positive for MOG antibodies has not been fully elucidated yet. It is important to investigate whether OCBs are simply a biomarker suggesting MS or whether inflammation in the CNS can also be a factor affecting the pathogenesis and prognosis in MOGAD. Therefore, we studied patients with MOG antibodies who tested positive for OCBs.

## 2 Methods

This was a single-center, retrospective, observational study. The participants were Japanese individuals >18 years of age, who visited Juntendo University Hospital between January and December 2022, presented with CNS symptoms (including optic neuritis), and were MOG-IgG-seropositive. Cerebrospinal fluids (CSF) samples were collected from all the patients *via* lumbar puncture. Patients were divided into the OCB-positive and negative groups, and the demographic (sex, onset age, disease duration, EDSS, lesion, clinical course, MOGAD criteria application), clinical laboratory (CSF cell count, protein level, IgG index, myelin basic protein level), and magnetic resonance imaging (MRI) data (MS MRI criteria (4) application, i.e. One or more T2-hyperintense lesions that are characteristic of MS in two or more of four areas of the CNS: periventricular, cortical or juxtacortical, and infratentorial brain regions, and the spinal cord; presence of a longitudinally extensive lesion over 3 vertebral segments) were compared between the two study clusters. The participants were evaluated based on the MOGAD diagnostic criteria (1). The studies involving human participants were reviewed and approved by ethics committee guidelines of Juntendo University (No. 2016014). Written informed consent to participate in this study was provided by the participants.

Cell-based assay (CBA) for MOG antibody detection was performed by an external laboratory (Cosmic Corporation, Tokyo, Japan) according to a previously described method (8). Isoelectric focusing and subsequent immunoblotting (LSI Medience, Tokyo, Japan) were used to determine OCBs. Student’s t-test for two samples was used to compare the continuous variables, whereas Fisher’s exact test was used to measure the frequency of the disease course (relapsing versus monophasic).

## 3 Results

**Figure 1:**
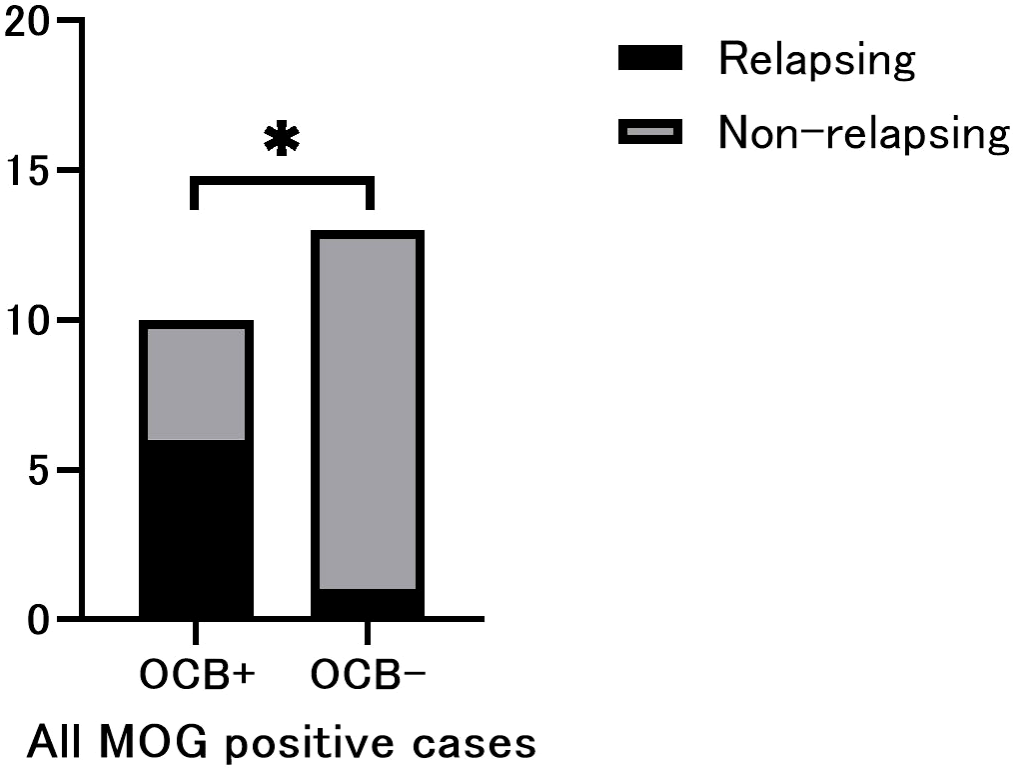
Phenotypic comparison between oligoclonal band (OCB)-positive and OCB-negative MOG positive cases. Relapsing cases were significantly more frequent in OCB-positive patients. Fisher’s exact test (n =23). *p < 0.05.

**Figure 2:**
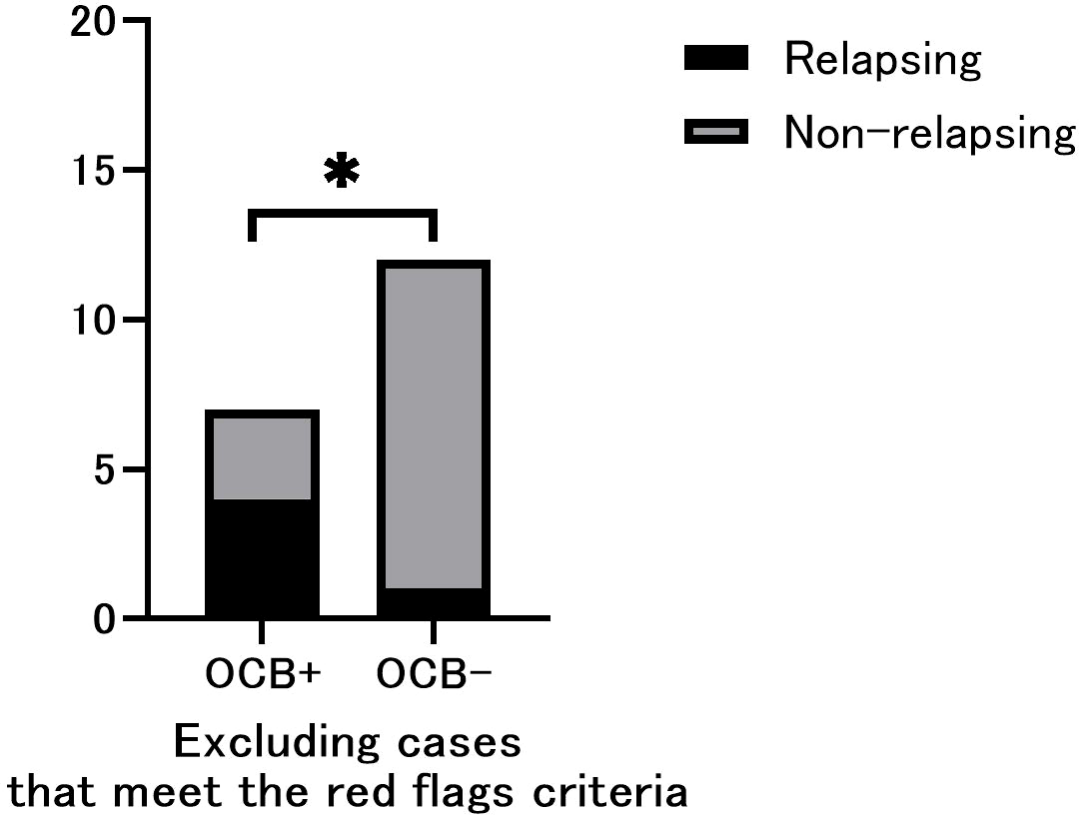
Phenotypic comparison between oligoclonal band (OCB)-positive and OCB-negative only in cases that do not include red flags. Relapsing cases were significantly more frequent in OCB-positive patients. Fisher’s exact test (n =19). *p < 0.05.

**Figure 3:**
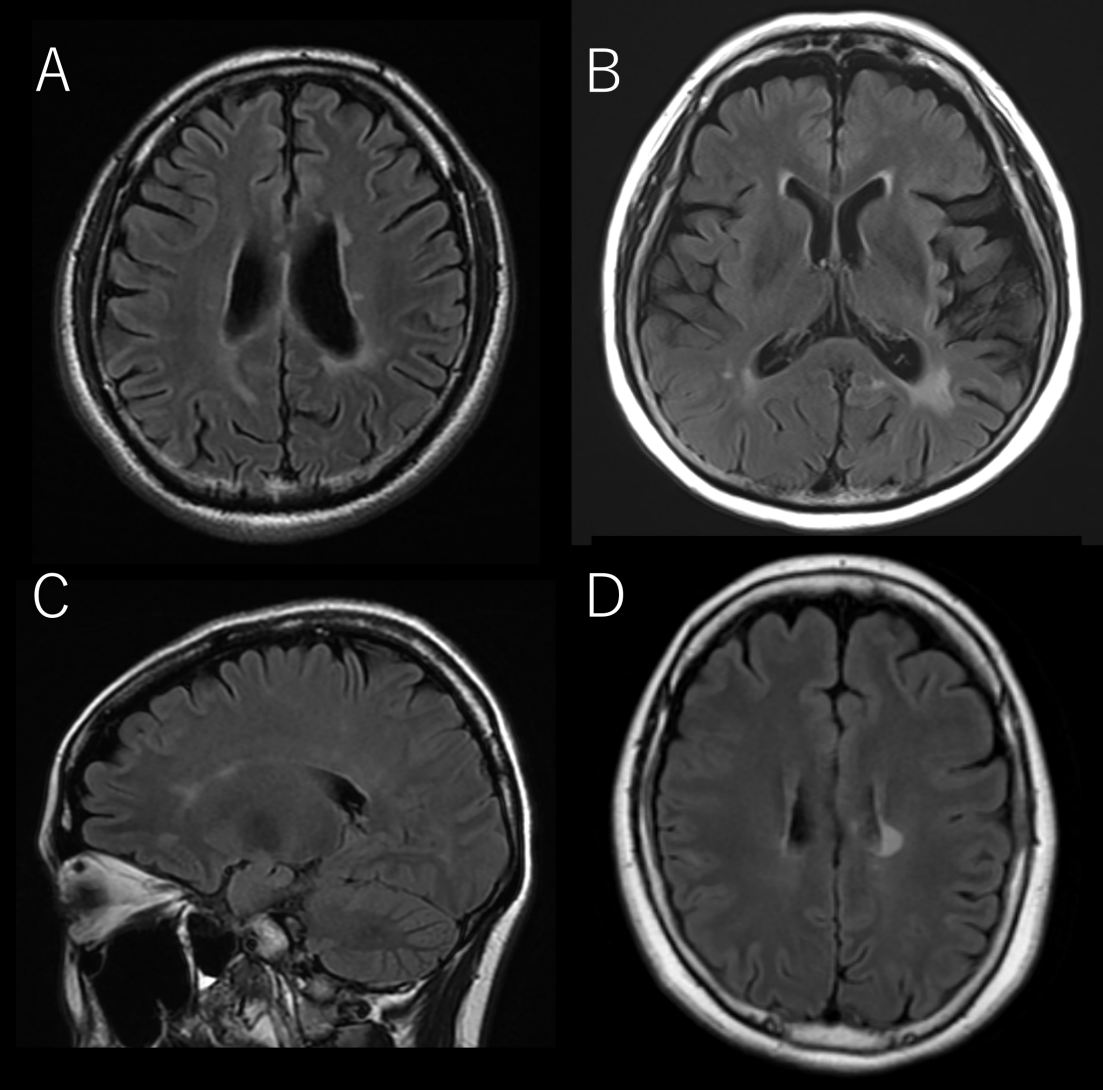
The representative brain MRI of cases that corresponded to the Red Flags indicated in **Table 4**. **(A)** OCB-negative-13, **(B)** OCB-positive-8, **(C)** OCB-positive-9, **(D)** OCB-positive-10.

A total of 23 patients met the selection criteria. Among them, 10 were OCB-positive and 13 were OCB-negative. Baseline patient characteristics such as sex, age at onset, disease duration, and Expanded Disability Status Scale (EDSS) scores (9) were not significantly different between the two patient groups. All the cases met the supportive criteria for MOGAD diagnosis. However, three positive cases also met the spatial dissemination criteria for MS diagnosis on MRI (4), and were classified as red flags. Additionally, one case each from OCB-positive and negative groups showed disease progression and met the red-flag criteria (**Table 1**). Notably, OCB-positive patients showed a significantly higher frequency of relapse (p=0.0186; **Figure 1**); and a significantly higher IgG index (p=0.0381; **Table 2**). The difference in relapse frequency regarding OCB status was also observed in the examination limited to cases meeting the diagnostic criteria for MOGAD (p=0.0379; **Figure 2**). The band number of oligoclonal bands was 5.3 ± 2.1. No significant differences in the MRI criteria for dissemination in space of MS were observed between the MOG antibody-positive and negative patients (**Table 3**). **Table 4** describes the conformity of specific diagnostic criteria for each case, while **Figure 3** displays the representative brain MRI scans of cases that corresponded to the Red Flags indicated in **Table 4**.

**Table 1:** Patient characteristics and OCB status.

OCB	Positive n=10	%, SD	Negative n=13	%, SD	Total n=23	%, SD
Female	8	80%	8	62%	16	67%
Onset age	32.0	± 9.8	42.8	± 15.4	38.1	± 14.3
Disease duration (years)	5.9	± 5.0	9.0	± 13.2	7.6	10.5
EDSS final visit	2.5	± 3.0	2.2	± 2.5	2.4	± 2.7
EDSSworst	4.0	± 2.3	3.9	± 2.1	3.9	± 2.2
Lesion						
Optic nerve	4	40%	9	69%	13	57%
Brain	7	70%	8	62%	15	65%
Spinal cord	4	40%	8	62%	12	52%
Clinical course						
Monophasic	3	30%	11	84%	14	61%
Relapsing	6	60%	1	8%	7	30%
Progressive	1	10%	1	8%	2	9%
Meet the red flags (1)	3/10	30%	1/13	8%	4/23	17%

OCB, oligoclonal band; SD, standard deviation; EDSS, expanded disability severity scale.

**Table 2:** OCB status and CSF parameters.

OCB	Positive n=10	SD	Negative n=13	SD	p value	Total n=23	SD
CSF cells (/μL)	18.8	± 13.9	29.2	± 74.0	NS	25.3	± 58.2
CSF protein (mg/dL)	32.0	± 9.8	42.8	± 15.4	NS	38.1	± 14.3
IgG index	0.75	± 0.26	0.57	± 0.83	<0.05	0.63	± 0.19
MBP (pg/mL)	442.6	± 718.0	227.3	± 559.1	NS	229.0	± 628.7

OCB, oligoclonal band; SD, standard deviation; CSF, cerebrospinal fluid; NS, not significant; MBP, myelin basic protein.

**Table 3:** OCB status and Spatial dissemination in MRI is a criterion for the diagnosis of MS.

OCB	Positive n=10	%	Negative n=13	%	Total n=23	%
Periventricular	4	40%	3	40%	7	30%
Infratentorial	4	40%	4	40%	8	35%
Spinal cord	4	40%	7	40%	11	48%
Cortical, juxtacortical	4	40%	3	50%	7	30%
Meets 2 or more criteria	3	30%	5	38%	8	35%
LESCL	3	30%	3	23%	6	26%

OCB, oligoclonal band; MS, multiple sclerosis; LESCL, longitudinally extensive spinal cord lesion over 3 vertebral segments.

**Table 4:** Conformity of specific diagnostic criteria.

OCB	Initial symptom	Supporting clinical or MRI features	Red flags	Dissemination in space by MRI for MS				
				Periventricular	Cortical or juxtacortical	Infratentorial	Spinal cord	two or more
(-)1	Optic neuritis	Optic neuritis; bilateral simultaneous clinical involvement		–	–	–	–	–
(-)2	Optic neuritis	Optic neuritis; bilateral simultaneous clinical involvement		–	–	–	+	–
(-)3	Optic neuritis	Optic neuritis; bilateral simultaneous clinical involvement		–	–	–	+	–
(-)4	Optic neuritis	Optic neuritis; longitudinal optic nerve involvement		–	–	–	–	–
(-)5	Myelitis	Myelitis; longitudinally extensive myelitis		–	–	–	+	–
(-)6	Optic neuritis	Optic neuritis; bilateral simultaneous clinical involvement		–	–	+	–	–
(-)7	Optic neuritis	Optic neuritis; longitudinal optic nerve involvement		–	–	+	–	–
(-)8	Optic neuritis	Optic neuritis; perineural optic sheath enhancement		–	–	–	–	–
(-)9	Optic neuritis	Optic neuritis; longitudinal optic nerve involvement		–	–	–	–	–
(-)10	Myelitis	Myelitis; longitudinally extensive myelitis		–	–	–	+	–
(-)11	Myelitis	Optic neuritis; bilateral simultaneous clinical involvement		–	–	–	–	–
(-)12	Optic neuritis	Optic neuritis; bilateral simultaneous clinical involvement		–	–	–	–	–
(-)13	Optic neuritis	Myelitis; H-sign	Progressive course*	+	+	+	+	+
(+)1	Brainstem	Ill-defined T2-hyperintensity involving pons		–	–	–	–	–
(+)2	Epilepsy	Cortical lesion		–	+	–	–	–
(+)3	Optic neuritis	Myelitis; H-sign		–	–	–	–	–
(+)4	Optic neuritis	Optic neuritis; perineural optic sheath enhancement		–	+	–	–	–
(+)5	Myelitis	Myelitis; conus lesion		–	–	–	+	–
(+)6	Myelitis	Myelitis; H-sign		–	–	–	+	–
(+)7	Brainstem	Cortical lesion		–	–	+	–	–
(+)8	Myelitis	Cortical lesion	DIS in MS-MRI criteria*	–	+	–	+	+
(+)9	Myelitis	Myelitis; Central cord lesion	DIS in MS-MRI criteria*	+	–	–	+	+
(+)10	Myelitis	Myelitis; conus lesion	Progressive course, DIS in MRI*	+	+	+	+	+

OCB, oligoclonal band; DIS, dissemination in space; MS, multiple sclerosis. *The representative MRI findings are presented in **Figure 3**.

## 4 Discussion

This study is the first to examine clinical differences based on the presence of OCBs in patients with MOG antibody-positive CNS inflammatory diseases. Notably, the OCB-positive cases had a high recurrence rate. However, as per the proposed diagnostic criteria, OCB-positive cases that met the dissemination in space criteria on MRI were considered as red flags of MOGAD. All of the cases in our cohort met the supportive criteria; however, three cases in the OCB-positive group showed spatially disseminated lesions on MRI that met the diagnostic criteria for MS, and hence were considered red flags. Additionally, one case in each of the OCB-positive and negative groups had a progressive course (OCB-positive case also met the MS-MRI feature) that also met the red flags. Therefore, it is highly likely that MS was included in the OCB-positive cases of this study. However, OCB-positive cases are common among patients who meet the diagnostic criteria for MOGAD. In addition, a significant tendency for recurrence was observed in OCB-positive cases, specifically limited to examples thIn addition, we observed a significant tendency for recurrence in OCB-positive cases, but only in cases that do not include red flags. Furthermore, there was no significant difference between the OCB-positive and OCB-negative cases in meeting the MRI spatial dissemination criteria for MS. The frequency of LESCL, which is rare in MS and frequently observed in neuromyelitis optica spectrum disorder (NMOSD) (10) and MOGAD, was not different between OCB-positive and OCB-negative groups, suggesting that the presence of OCBs does not affect the pathophysiology of myelitis. In the differentiation of MOGAD from MS and NMOSD, Cortese et al. have presented useful information based on a large database (11–13).

Gastaldi et al. reported that persistent MOG-IgG seropositivity and high remission titers were associated with an increased risk of relapse (14), identifying them as risk factors for MOGAD recurrence. Satukijchai et al. reported that an initial attack of optic neuritis is also a risk factor for MOGAD relapse (15). In our cohort, no significant differences in lesion distribution were observed between the OCB-positive and negative groups. Huda et al. reported that steroid treatment for more than one month decreased the relapse rate (16). Chen et al. reported that IVIg treatment effectively reduces the relapse rate of MOGAD (17). However, neither report considered the influence of OCB on relapse rate. In accordance with the treatment protocol for NMOSD, we followed maintenance steroid therapy in our study, regardless of the presence of OCB; therefore, we determined that any difference in treatment would not affect the outcome of this study. The presence of OCB is reported to be a poor prognostic factor for MS (6, 7). Although there is limited mention of the clinical implications of OCB positivity in MOGAD, Cobo-Calvo et al. found an association between OCB positivity and relapsing transverse myelitis, as well as the MS-like/optico-spinal phenotype (18).

A limitation of this study is that it was conducted at a single center with a relatively small number of cases. Since we did not match the background due to the rarity of the disease, there is a possibility that it may have affected the results of this study. Although the MOG antibody measurement method used in this study is included in the MOGAD diagnostic criteria, the lack of antibody titers may have affected the research results. The high prevalence of OCB-positivity suggests that it may not accurately reflect the overall pathology of MOGAD.

In conclusion, MOG antibody-positive cases with OCB positivity require careful treatment decision-making, i.e., whether to administer more potent classical immunosuppressive therapy or to initiate disease-modifying therapy for MS. Borderline cases between MOGAD and MS are often encountered in daily clinical practice. The new MOGAD diagnostic criteria have raised awareness of the need to exclude MS in diagnosis. However, there is still insufficient data to say that all cases that meet the red flags are MOGAD and not MS. Similarly, MS can also exhibit spinal cord lesions characteristic of MOGAD. In this study, true MOGAD was considered to be included in MOG-positive and OCB-positive cases that met the red flags of the MOGAD criteria. It was considered that detecting such cases in the future would be a challenge. Moreover, MS is a heterogeneous group of diseases without specific biomarkers, and examining the group of cases with positive MOG antibodies and positive OCBs may lead to elucidation of the pathophysiology of inflammatory diseases of the CNS, including MS, as a spectrum. This study is a retrospective study of a small number of cases, and it is insufficient to draw conclusions as scientific data. Further accumulation of cases is necessary.

## Data availability statement

The raw data supporting the conclusions of this article will be made available by the authors, without undue reservation.

## Ethics statement

The studies involving human participants were reviewed and approved by ethics committee guidelines of Juntendo University (No. 2016014). Written informed consent to participate in this study was provided by the participants.

## Author contributions

YT contributed in conception and design of the study and acquisition of data. YH, RK, KY, YT, DC, and NH performed the analysis and interpretation of data. YT wrote the first draft of the manuscript. All authors contributed to the article and approved the submitted version.
